# Improvement of Fast Model-Based Acceleration of Parameter Look-Locker T_1_ Mapping

**DOI:** 10.3390/s19245371

**Published:** 2019-12-05

**Authors:** Michał Staniszewski, Uwe Klose

**Affiliations:** 1 Institute of Informatics, Silesian University of Technology, Gliwice 44-100, Poland; 2 Department of Diagnostic and Interventional Neuroradiology, Eberhard Karls University, Tübingen 72076, Germany; Uwe.Klose@med.uni-tuebingen.de

**Keywords:** constrained and sparsity reconstruction, model-based approach, inversion-recovery Look-Locker, undersampled T_1_ mapping, optimization, medical imaging

## Abstract

Quantitative mapping is desirable in many scientific and clinical magneric resonance imaging (MRI) applications. Recent inverse recovery-look locker sequence enables single-shot T_1_ mapping with a time of a few seconds but the main computational load is directed into offline reconstruction, which can take from several minutes up to few hours. In this study we proposed improvement of model-based approach for T_1_-mapping by introduction of two steps fitting procedure. We provided analysis of further reduction of k-space data, which lead us to decrease of computational time and perform simulation of multi-slice development. The region of interest (ROI) analysis of human brain measurements with two different initial models shows that the differences between mean values with respect to a reference approach are in white matter—0.3% and 1.1%, grey matter—0.4% and 1.78% and cerebrospinal fluid—2.8% and 11.1% respectively. With further improvements we were able to decrease the time of computational of single slice to 6.5 min and 23.5 min for different initial models, which has been already not achieved by any other algorithm. In result we obtained an accelerated novel method of model-based image reconstruction in which single iteration can be performed within few seconds on home computer.

## 1. Introduction

In clinical routines, application of magnetic resonance (MR) parameters proton density and the relaxation times T_1_ and T_2_ lead to distinction of different physical tissues in parameter weighted images. These images provide only qualitative data. Quantitative evaluation such as T_1_ mapping, however, can give directly properties of tissues, which are independent from technical impacts. This approach offers a better comparison of different patients across different scanners, and enables classification of diseases and further analysis of pathological processes [1]. Thus, quantitative mapping is desirable in scientific and clinical MRI applications for brain studies, myocardial, T_2_-mapping and dynamic studies [2,3]. In conventional acquisition, especially T_1_ mapping, suffers from long scan time and restricted spatial resolution with limited T_1_ accuracy. In current practice, to face with the problem of measurement time, acquisition sequence is based on the Look-Locker (LL) concept [4,5] with former application of inversion recovery (IR) pulse and continuous readouts Steady-State Free Precession (SSFP) or Fast Low Angle Shot (FLASH). Recent IR-LL sequence enables single-shot T_1_ mapping with time of few seconds but in that case the main computational load is directed into reconstruction procedure, which can take from several minutes up to few hours. Hence the improvement of image reconstruction along with optimization of methods is of high interest.

In the model-based approach [6,7,8,9,10,11,12] the parameter maps are estimated directly from the undersampled k-space by iterative reinserting original k-space and model parameters fitting. Tran-Gia et al. worked on model-based methods in his publications proposing pixel-wise fitting of T_1_^*^ and M_0_^*^ [13] and dictionary-based approach for T_1_-mapping [14,15]. These effective parameters describe the T_1_ relaxation process under the influence of repetitive small-angle excitations, which are necessary to observe the relaxation process after a single inversion pulse. The proposed algorithms require many iterations of fitting and computational time is higher than one hour for a single slice on a home computer without central processing unit (CPU) and graphics processing unit (GPU) acceleration. The series of different methods were presented by Wang et al. in the form of regularized nonlinear inversion (NLINV), conjugate gradient (CG) along with pixel-wise fitting [16,17], the iterative regularized Gauss–Newton method (IRGNM) [18], simultaneous estimation of all parameters, L_1_ regularization and the fast iterative shrinkage-thresholding algorithm (FISTA) [19]. In that case, despite application of external libraries and GPU acceleration, the fastest offline calculations are still performed in 10–20 min and even more.

In order to reconstruct undersampled k-space data the methods of compressed sensing (CS) can be applied, which relies on the idea of sparsity of MR images. Further speed improvement may be achieved by combination of CS with parallel imaging, which has been already used in parametric mapping [20,21,22,23,24,25,26]. Zibetti et al. provides comparison and evaluation of 12 different types of CS sparsity for acceleration of T_1_ mapping [27]. More recent methods use improvements of previous algorithms by means of total-generalized-variations (TGV) based regularization and further adapted to a multiparametric setting [28] or split-slice GRAPPA and a model-based iterative algorithm for T_2_-mapping [29]. Different approach bases on the method of magnetic resonance fingerprinting [30,31] in which the benefit comes from simultaneous computation of T_1_ and T_2_ maps but in slightly longer acquisition time. Other methods base on under-sampled k-t space data but used mainly in cardiac application in observation of periodic changes of dynamic heart data [32]. Most of the current works operates on a single slice, which from a clinical point of view is not applicable. The limited methods of multi-slice parameter mapping have been used in few works [28,29,33] resulting in calculation time from 10 min [28] up to 7 h [29] in multi-slice analysis. In multi-channel systems the number of coils may be limited in the preprocessing step. The coil compression can be applied by singular value decomposition [34] or by evaluation of virtual channels using a principle component analysis [18,19,33].

Application of more complex methods implies efficient results but also increases the computational time showing a request for a simpler approach (such as the fitting method) with satisfactory efficiency but faster calculation time. In this study we proposed an improvement of a model-based approach for T_1_-mapping by introduction of a two steps fitting procedure, which for the purpose of that work we called fast inversion recovery Model-based Acceleration of Parameter mapping (FIR-MAP). This approach has one strong advantage relying on time acquisition of 6 s as well as an effective and fast fitting procedure that shortened the time of evaluation. We verified two different initial models and applied the analysis of further k-space data reduction, which lead us to decrease of computational time and to perform a simulation of multi-slice development. In result we obtained an accelerated novel method of model-based image reconstruction in which a single iteration can be performed within a few seconds on a home computer. Finally, the FIR-MAP method was compared to the IR-MAP [14] and reference segmented data basing on in vivo human brain measurements. Along with that work we provide Matlab source code in the Supplementary Materials.

## 2. Materials and Methods

### 2.1. Original Data

Original data were taken from available online source provided by Tran-Gia under [35]. All measurements were performed on a 3T whole-body scanner (MAGNETOM Trio, Siemens AG Healthcare Sector, Erlangen, Germany) applying a 12-channel phased-array head coil. The studies were performed with an inversion-recovery Look-Locker (IR-LL) sequence in order to obtain T_1_ measurements. Obtained T_1_ values were evaluated in the ROIs containing white matter (WM), grey matter (GM) and cerebrospinal fluid (CSF). The dataset consists of:

1. In-vivo studies of the brains of seven healthy volunteers (aged between 23 and 30 years) for field of view (FoV) ranging from 200 × 200 to 220 × 220 mm^2^, slice thickness: 4 mm, T_E_ = 2.5 ms, T_R_ = 6 ms, flip angle: 7°, total time of scan 6 s with a golden ratio radial k-space trajectory.
2. Additionally, a fully sampled IR-LL dataset of single 2D slice was acquired using the segmented process in order to obtain reference data. A single acquisition of one segment (single IR-LL measurements) took 6 s (each of which was followed by a 15 s break) and was repeated 100 times in order to fulfill k-space with single lines of data. For in vivo measurements a total time scan was reduced to approximately 30 min.

Acquired data is organized in following way:

- np—number of projections (i.e., 999 original projections),
- nc—number of coils (i.e., four coils covering whole head for each projection),
- nr—number of readout points (i.e., 256 points given in k-space for each coil and projection).

### 2.2. Hardware Specification

The base for comparison of the proposed FIR-MAP method was the results obtained from IR-LL segmented data treated in the original work as a reference (REF) and the IR-MAP algorithm by Tran-Gia [14], which is the map acceleration method for the interpolated first model (IFM). All calculations were performed in Matlab (The MathWorks, Natick, MA, USA) on two different home computers 2.6 GHz Inter Core i7, 16 GB RAM and 3.5 GHz 6-Core Intel Xeon E5, 64 GB RAM without any GPU acceleration, using standard Matlab libraries and six workers.

### 2.3. Processing Scheme

**Figure 1:**
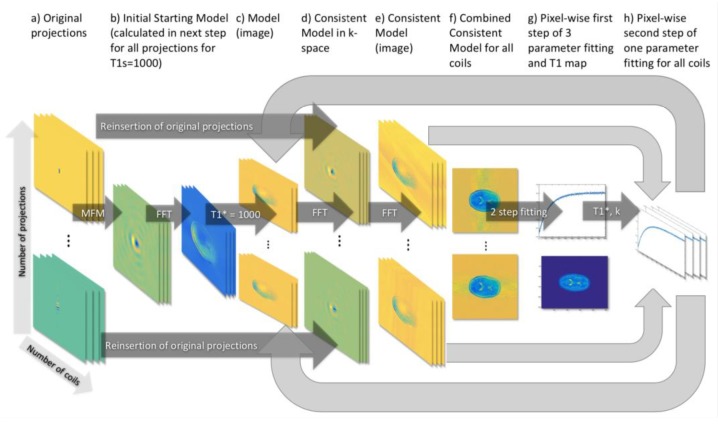
General scheme of the fast IR-MAP (FIR-MAP) proposed for acceleration of the IR-MAP. Each step of the algorithm is placed in separated column from (a) to (h). Multiple rows (two rows with dots) correspond to multiple projections that can be present in dataset (a single row is understood as a combined image for all projections—in that case MFM—mean first model). In general, each projection consists of multiple coils (one image after another) and in f) all coils are combined for all coils resulting in single image for all projections.

In order to match radial sampling scheme, np trajectories were generated with the golden ratio [36] radial profile order. Having radial trajectories, the nr readout points were inserted into a Cartesian grid for each projection np using GROG operation [37,38]. Original projections (radial k-space data inserted into a Cartesian grid using GROG operation) were not modified across the whole algorithm and in the reinsertion process might be used without any modifications or by taking projections fulfilling sparsity condition, which slightly improves results. In sparsity case k-space was calculated from a fully sampled image obtained from the last 200 projections of the IR-LL measurement. The following steps were performed in the FIR-MAP in the reconstruction scheme presented on Figure 1:

1. Original projections (a) for all coils were used to create the initial starting model (b) for which T_1_^*^ was assumed to be equal for the sake of simplicity at the beginning.
2. The model (as imaged) was created for all coils and projections (c).
3. The consistent model (d) was generated by taking Fourier transformation (FT) of the initial starting model (b) and reinsertion of the original projections (a) in k-space. The consistent model (e) in the next steps was used in the image space.
4. The first part of pixel-wise fitting (g) was performed on the consistent model for each projection combined for all coils (f).
5. The second part of pixel-wise fitting (h) bases on the consistent model for each projection and for each coil (e) and the results of the first part of fitting (g).
6. The iteration process repeats again from evaluation of model (c).

#### 2.3.1. Initial Starting Model for First Iteration of the FIR-MAP

The first step in the FIR-MAP algorithm can take three possible initial models given to the first iteration:

1. OFM—original first model - original projections in the Cartesian grid in the first model [13], which due to the high number of required iterations were skipped in this study,
2. IFM—interpolated first model of all acquired k-spaces points through time [14] by performing a linear interpolation in order to improve convergence of incomplete k-space not covered by any data points,
3. MFM—our proposition—mean first model, which is calculated by taking all original projections. In the evaluation of MFM only non-zero values are taken to the mean for the resulting k-space for each coil (points that are not covered by any projections are not taken to the mean). After FT the combined image for each coil is treated as $M_{0}^{*}$ in simplified formula for $T_{1}^{*}$ = 1000:
  (1) $$M_{\mathrm{MFM}}\left(t\right)=M_{0}^{*}\left(1-2e^{\frac{-t}{T_{1}^{*}}}\right).$$

#### 2.3.2. Consistent Model, Termination Criterion and Coil Combination

The iteration process starts in the next step. The model has to be generated for each inversion time (projection) and for each coil. In that step all original projections are reinserted to the initial model and the circular k-space mask can be applied. The absolute sum of difference of original k-spaces and reconstructed k-spaces was proposed for the termination criterion as well as in observing the reconstructing progress. The fixed number of iterations might be also assumed. Combined data $M_{comb}\left(j,t\right)\;$ for each coil nc and each pixel *j* was calculated with application of meanPhase map $\varphi_{nc}\left(j\right)$ [14] taken at the beginning from a fully sampled image obtained from the last 200 projections of IR-LL data and the complex-valued consistent model of current iteration $M_{nc}\left(j,t\right)$.

(2) $$\theta \left(j,t\right)=\underset{nc}{\sum }\left[sign\left(Real\left\{M_{nc}\left(j,t\right)*\;e^{-i\varphi_{nc}\left(j\right)}\right\}\right)*\;{\left|Real\left\{M_{nc}\left(j,t\right)*\;e^{-i\varphi_{nc}\left(j\right)}\right\}\right|}^{2})\right].$$

(3) $$M_{comb}\left(j,t\right)=sign\left(\theta \left(j,t\right)\right)\;\sqrt{\left|\theta \left(j,t\right)\right|}).$$

#### 2.3.3. Two Steps Fitting Procedure—the First Step

In the first step three parameters pixel-wise fitting was applied by the nonlinear regression using the specified model of relaxation process. The coefficients were estimated using iterative least squares estimation [39,40,41]. The initial guess of the fitting method was in the first iteration taken as a maximum value in magnetization curve for $M_{0}^{*}$ and a minimum value for M_0_. Each next iteration took values from a previous iteration as the initial guess. The fitted model is given by [42]:

(4) $$M\left(t\right)=M_{0}^{*}-\left(M_{0}+M_{0}^{*}\right)e^{\frac{-t}{T_{1}^{*}}}.$$

According to obtained parameters M_0_, $M_{0}^{*}$ and $T_{1}^{*}$ it is possible to calculate T_1_ by formula:

(5) $$T_{1}=T_{1}^{*}\frac{M_{0}}{M_{0}^{*}}.$$

#### 2.3.4. Two Steps Fitting Procedure—the Second Step

In the second step, in order to deal with influence of each coil, the model had to be refitted for each coil in separate iteration. $T_{1}^{*}$ could be taken from the first step of fit (for each coil it had the same value) and the factor k (7) was introduced in order to reduce fitting procedure to one parameter $M_{0}^{*}$ influenced by coils sensitivities. The fast one parameter linear fit could be performed by solving systems of linear equations for real and imaginary part separately:

(6) $$M{\left(t\right)}_{\mathrm{nc}}=M_{0}^{*}(1-\left(k+1\right))e^{\frac{-t}{T_{1}^{*}}},$$

where:

(7) $$k=\frac{M_{0}}{M_{0}^{*}},$$

and simplified:

(8) $$T_{1}=T_{1}^{*}k.$$

For fitted parameters the new model was generated and original data was again reinserted, which created the consistent model and the iteration process was repeated until reaching the termination criterion.

### 2.4. Reduced Number of Projections

In order to improve time complexity of the FIR-MAP method the reduction of number of projections can be performed. In such a case the first initial model is calculated by taking all original projections but the iteration process works with each nth projection resulting in less data analysis. In another case the reduced number of original projections is applied for both in the step of creation of the first model as well as in the iteration process.

## 3. Results

### 3.1. Single Slice Analysis with Total Number of Projections

**Figure 2:**
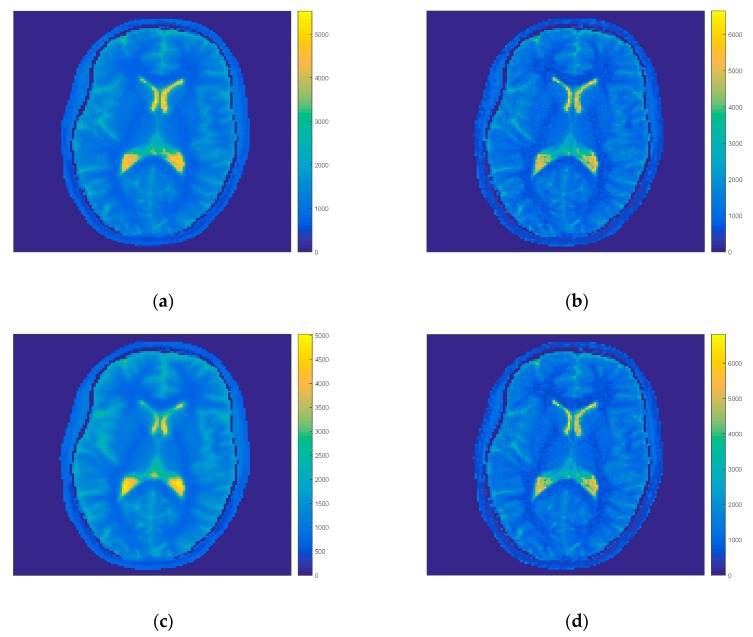
Exemplary results for volunteer V3 of T_1_ map estimation with (1) our initial model FIR-MAP-MFM (**a**) after termination of 150 iterations and (2) interpolated model FIR-MAP-interpolated first model (IFM; **b**) after termination of 30 iterations and corresponding results for reference methods REF (**c**) and IR-MAP (**d**). The FIR-MAP-MFM (a) gives similar T_1_ map to the REF method (c), while the higher spatial resolution can be observed for the FIR-MAP-IFM (b) and the IR-MAP (d).

**Figure 3:**
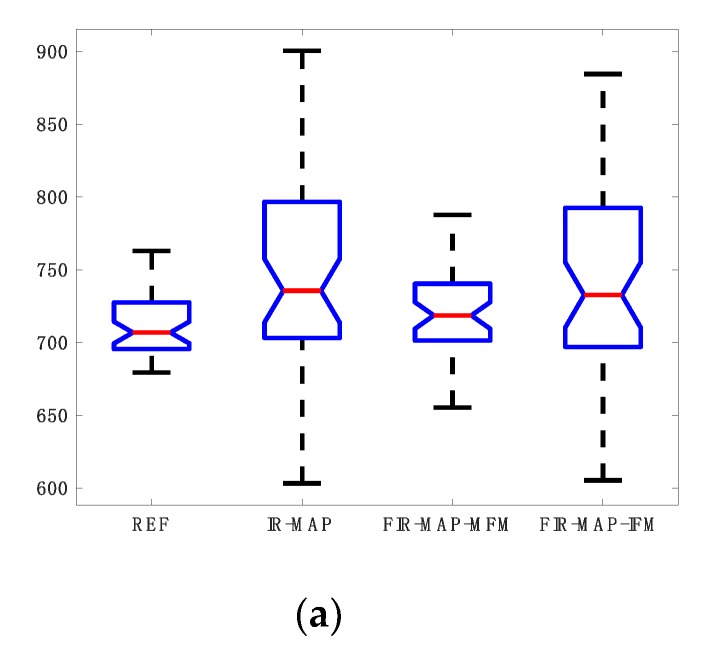

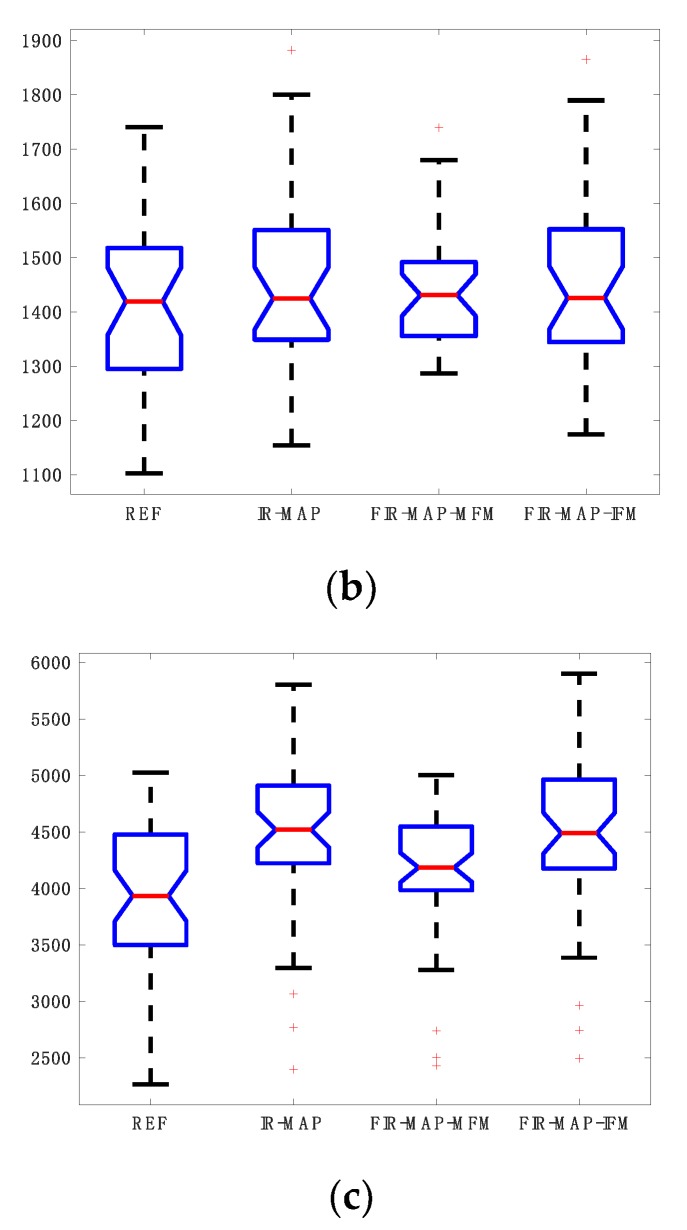
Exemplary results of ROI analysis for white matter (WM; **a**), grey matter (GM; **b**) and cerebrospinal fluid (CSF; **c**) in the form of boxplots for volunteer V3 of T_1_ map estimation for (1) our initial model MFM (FIR-MAP-MFM) after termination of 150 iterations and (2) interpolated model IFM (FIR-MAP-IFM) after termination of 30 iterations and corresponding results for reference methods (REF and IR-MAP). The FIR-MAP with two different initial models (MFM and IFM) gives similar results to reference methods REF and IR-MAP.

In the first step of comparison all projections were taken for the first model and iteration process. The IR-MAP and REF were taken as reference results and our approach the FIR-MAP was tested for two cases (1) with our initial model MFM and (2) with interpolated model IFM. Results of reconstruction of the FIR-MAP with both initial models and the reference IR-MAP and REF can be observed in Figure 2 for volunteer V3. The ROI analysis of regions WM, GM and CSF are presented for all methods in the form of boxplots in Figure 3. The total number of iterations in first case was set to 150 and in second case to 30. Introducing IFM improved highly results of reconstruction and it could be observed that spatial resolution was still better for the IFM initial model, which was reported [14] explaining that the reason lay in only 6 s of acquisition (minimizing motion artifacts). On the other side mean values of T_1_ in selected ROI and its deviation was more accurate for MFM.

The results of ROI analysis for all volunteers are presented in Table 1 (and Table A1 in Appendix A) calculated for the FIR-MAP and reference methods (REF and the IR-MAP). Table 1 consists of numerical results of the FIR-MAP started with an MFM initial model ran for 150 iterations. The ROI analysis of the FIR-MAP with MFM shows that the differences between mean values with respect to the REF method were WM—0.3%, GM—0.4% and CSF—2.8%, which in comparison to the IR-MAP (WM—1.4%, GM—2.1% and CSF—11.3%) gave more stable results. The values of mean/std (the relation of mean value and standard deviation in selected ROI) were also better for FIR-MAP in comparison to IR-MAP and more comparable to the REF method. Table 1 presents also numerical ROI analysis of the FIR-MAP for IFM initial model calculated for 30 iterations. The results show that the differences of the FIR-MAP with respect to REF were WM—1.1% (IR-MAP 1.4%), GM—1.78% (2.1%) and CSF—11.1% (11.3%). For all regions the FIR-MAP gave slightly better results than the IR-MAP and the mean/std values were comparable. The advantage was that FIR-MAP gave such results faster—after 30 iterations (instead of 50 iterations of the IR-MAP).

### 3.2. Improvement of Single Slice Analysis

The implementation of FIR-MAP had reduced time complexity of single iteration without any loss in ROI quality. In general, single iteration of the FIR-MAP was completed in approximately 30 s for 999 projections, four coils and 256 image resolution. With 30 s:

1. The reinsertion of original data into the model and calculation of combination of all coils took 20 s.
2. The first step of 3-parameter fitting of model ran 5.5 s in parallel (20 s sequentially).
3. The second step of one parameter linearized fit required 4.5 s.

The only one part in which the parallel for loop was introduced was the place of three-parameter fitting, which took 5.5 s for six workers on a desktop computer. In contrast the same part run sequentially would take 20 s. The time complexity of the FIR-MAP can be decreased by taking each nth projection in the iterative reconstruction process. At the beginning after data acquisition all projections were taken in order to compute the (1) MFM model and (2) IFM model. However, in each iteration process each nth projection was used in reconstruction. Here some compromise should be achieved between decreasing quality of the ROI values and run time of a single iteration, which after each fifth projection changed slightly (Table 2).

Two initial models MFM and IFM were verified for the FIR-MAP in order to check the influence of taking each second and sixth projection in iteration process. For MFM (Table 3 and Table A2 in Appendix A) with higher reduction the loss in mean/std was increasing while the quality of T_1_ values in ROI analysis was decreasing. For the same data IFM (Table 3 and Table A2 in Appendix A) showed slightly smaller changes. The reason of such situation lies in the way of evaluation of IFM, which after taking all projections in model generation required less iterations to get better results even if a smaller number of projections were taken to the reconstruction process. In this situation the iteration process influences mainly the values of single pixels and not the structure and resolution.

**Figure 4:**
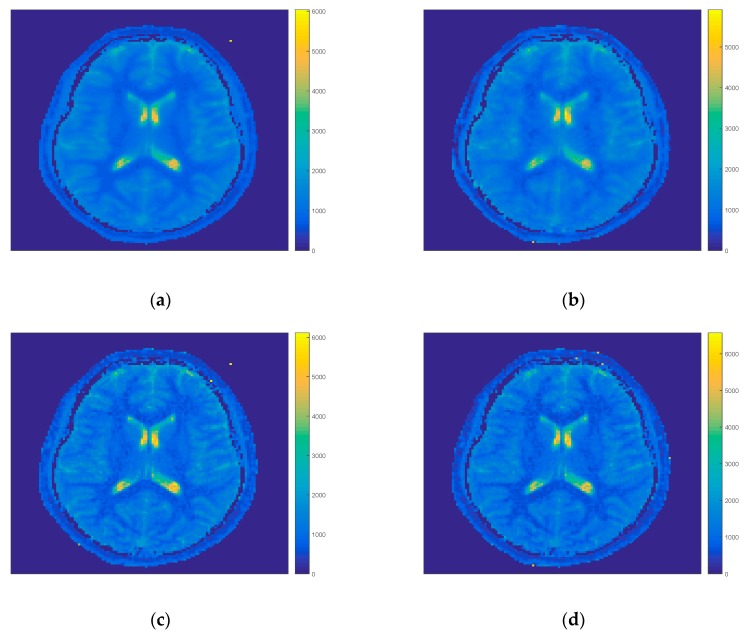
T_1_ maps results of FIR-MAP for MFM for each projection (**a**), for each sixth projection (**b**), IFM for each projection (**c**) and by taking each sixth projection (**d**) in the iteration process for volunteer V2. The spatial resolution and image quality decreases for both initial models for each sixth projection (right column **b** and **d**) with respect to each projection (left column **a** and **c**).

Figure 4 shows the influence of further reduction of projections on the quality of T_1_ maps. By decreasing the number of projections taken to the iteration process the quality of the FIR-MAP with MFM decreased, however, by considering only each sixth projection the contrast and resolution was comparable to the reference results. For IFM the spatial resolution was still better due to the way of evaluation of model for all projections. Due to that feature it was possible to decrease the time of computation of single iteration even for 9 s (for each sixth projection).

### 3.3. Simulation of Using a Reduced Number of Projections

**Figure 5:**
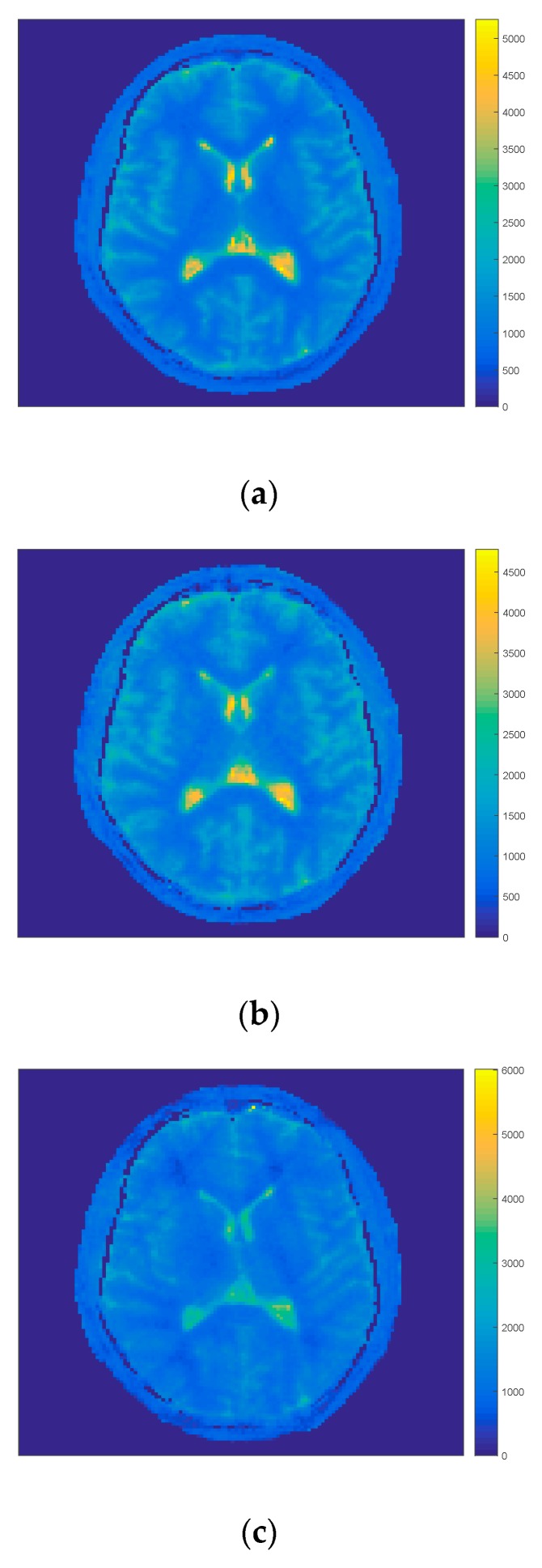
T_1_ maps of FIR-MAP for MFM taking each second (**a**), sixth (**b**) and seventh (**c**) projection in initial step and iteration process for volunteer V7. The spatial resolution and image quality decreases with the number of projections (from **a** to **c**).

It was shown that an appropriate T_1_ evaluation was possible in reduced processing time using a strongly reduced number of projections, while the initial model was still calculated using all projections. If a reduction of projections was also possible in this first part of the data processing, the acquisition of the skipped projections could be omitted and data from parallel slices could be acquired in this time. A multi slice measurement in only 6 s would be then possible. For the IFM model the reconstruction scheme and evaluation of first model was possible for up to each second projection—for a higher number the initial model was noisy and the reconstruction generated a lower value of the mean/std. The power of the IFM model was connected to the number of points for which the interpolation could be performed. In the case of decreasing the number of projections, the number of interpolated points was also limited and the higher spatial resolution, which was the main advantage of the approach was not visible. In contrast to IFM, application of our MFM model gave more promising T_1_ maps. Table 4 (and Table A3 in Appendix A) and Figure 5 show results of MFM with each second and sixth projection proving that even after taking each sixth projection it was possible to reconstruct the final T_1_ map. For each second projection the change in ROI values (WM—1.4%, GM—0.2% and CSF—1.38%) and mean/std was still comparable to reference data, for each sixth projection differences increased (WM—1.4%, GM—2.14% and CSF—6.24%) but the results were still comparable showing that it was possible to use the FIR-MAP for multi-slice of five simultaneous slices for 999 time stamps.

## 4. Discussion

### 4.1. Two Steps Fitting

**Figure A1:**
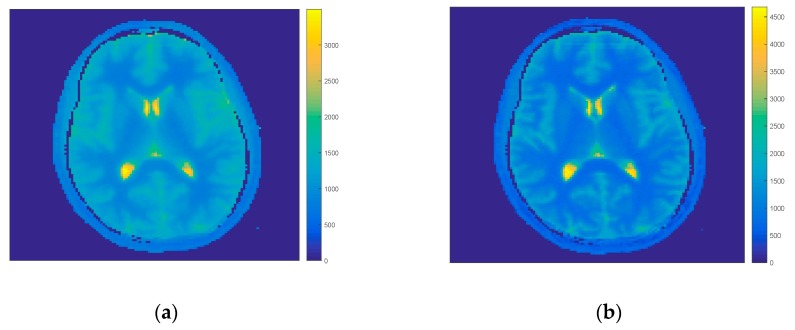

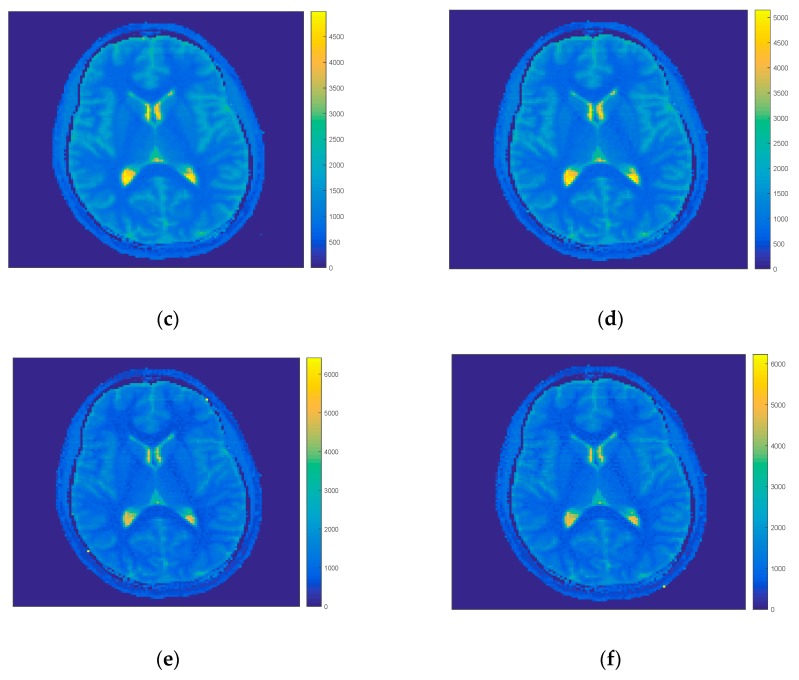
T_1_ maps of volunteer 1 calculated by the FIR-MAP-MFM after (**a**) 50, (**b**) 150, (**c**) 300, (**d**) 450, (**e**) 600 and (**f**) 750 numbers of iterations.

The original work of Tran-Gia et al. [14] deals with the time-consuming dictionary-based approach, which depends on the size of dictionary entries, instead of a mono-exponential fit. The iterative fitting procedure was highly improved by application of interpolation within undersampled original data. In contrast in our work we proposed a two steps model fitting—in the first step using the nonlinear regression we were able to fit the T_1_ relaxation curve for the combined image while in the second step we applied a time efficient linear fit in order to calculate the M_0_ values weighted by the coil sensitivities. The benefit of this approach is that there is no additional need of measurement in which coil sensitivities will be evaluated and the coil influence is updated in each iteration ensuring correctness of data. Such a procedure allowed us to decrease run time and obtain comparable T_1_ values. With this two steps fitting procedure of the FIR-MAP we were able, by mono-exponential fit, to obtain similar T_1_ values in selected ROI to the reference IR-MAP. In the FIR-MAP we used two initial models basing on the idea of projection interpolation (IFM) proposed by Tran-Gia et al. [14] and our proposition based on the mean of projection (MFM). The IR-MAP algorithm was able to finish reconstruction within 50 iterations while in contrast our FIR-MAP with IFM could finish with similar T_1_ values after 30 iterations. The disadvantage of IFM was related also to some outstanding data, which was present due to imperfection of the interpolation process and a lack of undersampled projections. Application of the MFM model requires more iterations (we used 150) but it has one strong advantage—by further reduction of the number of projections, the comparable T_1_ values in selected ROI are still possible after taking each sixth projection, which will be useful in further multi-slice analysis for 999 time stamps. The FIR-MAP-MFM gives T_1_ values in selected structures with lower resolution but not worse than the REF method, while the higher spatial resolution can be observed for the FIR-MAP-IFM and the IR-MAP. The spatial resolution of the FIR-MAP-MFM can be improved by application of a higher number of iterations (Figure A1 in Appendix A). The advantage of T_1_ maps in clinical application lies in the possibility of tissue characterization within a certain region of interest and not in discrimination between tissues. Therefore, our intension was to apply T_1_ maps and observe the influence of algorithm on mean values and standard deviations of selected ROI. Other work [33] assumes 1492 time stamps—which could in future increase further undersampling.

### 4.2. Time Complexity

Tran-Gia et al. [14] showed that undersampled data can be acquired within 6 s due to the IR-LL sequence but the reconstruction algorithms require minutes and hours to obtain full results. In our work we stated two main assumptions—(1) to improve computational time of image reconstruction by decreasing the number of iterations and run time of single iteration and (2) to obtain comparable quality of T_1_ maps by means of ROI analysis. The FIR-MAP—our contribution—fulfilled those conditions. Few approaches in T_1_-mapping reconstruction have been already presented. In order to get better time of computational we decided to choose a simple and efficient method of model fitting. Other solutions, in some cases are more efficient but are also numerically advanced, and require more time to reconstruct the final image [16,17,18,19,27,28,29,33]. In the literature we did not find any benchmark that enables us to compare methods of T_1_ mapping reconstruction and quality of results of different works for similar data. Instead for that purpose, we used the available real data provided by Tran-Gia et al. along with the results of their work and reference data [14]. It was also difficult to analyze time complexity of other works due to different image resolution, number of coils and time stamps. The basic IR-MAP algorithm [14] requires approximately 100 s (reported 90 s for fitting procedure and remaining part for reinserting) to evaluate a single iteration, which for an assumed 50 iterations gives approximately 85 min of the whole iteration process and data preparation for a single slice. Acceleration using GPU and implementation of some methods in C/CUDA (Compute Unified Device Architecture) generates a time complexity of 10–20 min [19,33] and in the range from minutes to hours depending on data size [18]. Fast multi-slice method for T_2_ mapping [29] calculates 50 slices on an office computer within 7 h, which gives approximately 9 min per slice or alternatively rapid T_1_ quantification [28] reports reconstruction time of approximately 10 min per slice. We showed that a single iteration of the FIR-MAP for all projections could take 30 s. The data preparation of initial model for all projections took an additional 2 min for IFM and 1 min for MFM. In that case a full T_1_ map reconstruction of a single slice would take 17 min for IFM and 76 min for MFM in comparison to 85 min of the original IR-MAP. We proved also that in the iteration procedure it was possible to obtain comparable results of ROI and mean/std even by taking each sixth projection. With that assumption we were able to decrease the time of computational of single slice to 6.5 min for IFM and 23.5 min for MFM, which has not already been achieved by any other algorithm. All calculations of our method were performed on standard Matlab libraries without any GPU acceleration. The only one part of the FIR-MAP: the three-parameter fitting procedure was done in parallel on six workers by application of a home computer.

### 4.3. Further Development

The crucial point of the FIR-MAP stands in the initial model. We could observe that introduction of IFM gave from the beginning good starting points, which required 30 iterations to get acceptable ROI values. On the other hand, IFM suffered from a lack of a number of points given to the interpolation procedure, which eliminates that solution in further data reduction. MFM in our case was developed for a simplified model, which could be improved in future works. In our future works we planned sequence modification in order to collect multi-slice data as well as introduction of parallel imaging. Our code would be reimplemented in a more efficient environment with parallel and GPU acceleration.

## 5. Conclusions

In this work we introduced the FIR-MAP model-based reconstruction method based on IR-LL sequence. We proposed an efficient and faster two steps fitting procedure tested for two initial models—IFM and MFM. The validation of our method was performed on data available online for in vivo brain studies for seven healthy volunteers compared to a segmented inversion recovery T_1_ mapping experiment and the IR-MAP. In both cases we got similar T_1_ values to the reference methods within selected ROI and high improvement in run-time of single iteration. We analyzed further reduction of number of projections, which decreased computational time into 6.5 min in the best case. Promising results were obtained by reduction of considered projections for the T_1_ mapping, which will allow us to proceed to multi-slice measurements within 6 s measurement time.

## Figures and Tables

**Table 1:** Results of T_1_ values (in ms) of ROI analysis (WM, GM and CSF) of all volunteers for initial model MFM calculated for 150 iterations of FIR-MAP, IFM calculated for 30 iterations of FIR-MAP in comparison to reference methods (REF and IR-MAP). Each method consists of a mean value in ROI (mean) and standard deviation (std). Additionally, results of all volunteers are presented in the corresponding mean/std.

	REF			IR-MAP			FIR-MAP-MFM			FIR-MAP-IFM		
	WM	GM	CSF	WM	GM	CSF	WM	GM	CSF	WM	GM	CSF
Mean	712	1402	3908	722	1432	4351	710	1407	4016	720	1427	4340
Std	22	117	555	78	162	651	31	115	469	79	159	602
Mean/Std	32	12	7	9	9	7	23	12	9	9	9	7

**Table 2:** Mean run time of single iteration with respect to each n-projection.

**Number of Each n Projection**	1	2	3	4	5	6	7	8
**Mean Run Time of Single Iteration (s)**	30	17.5	13.3	10.6	9.5	9	8.3	8

**Table 3:** Results of T_1_ values (in ms) of ROI analysis for initial model MFM calculated for 150 iterations and initial model IFM calculated for 30 iterations of FIR-MAP by taking all projections (FIR-MAP-MFM, FIR-MAP-IFM), each second (FIR-MAP-MFM-2, FIR-MAP-IFM-2) and sixth (FIR-MAP-MFM-6, FIR-MAP-IFM-6) projection in iteration process. Each method consists of mean value in ROI (mean) and standard deviation (std). Additionally, results of all volunteers are presented in the form of mean/std.

	FIR-MAP-MFM			FIR-MAP-MFM-2			FIR-MAP-MFM-6		
	WM	GM	CSF	WM	GM	CSF	WM	GM	CSF
Mean	710	1407	4016	701	1402	3959	701	1370	3733
Std	31	115	469	41	115	459	68	128	408
Mean/Std	23	12	9	17	12	9	10	11	9
	**FIR-MAP-IFM**			**FIR-MAP-IFM-2**			**FIR-MAP-IFM-6**		
	**WM**	**GM**	**CSF**	**WM**	**GM**	**CSF**	**WM**	**GM**	**CSF**
Mean	720	1427	4340	717	1426	4332	710	1416	4309
Std	79	159	602	79	161	597	80	161	589
Mean/Std	9	9	7	9	9	7	9	9	7

**Table 4:** Results of T_1_ values (in ms) of ROI analysis for initial model MFM calculated for 150 iterations of the FIR-MAP by taking all projections (FIR-MAP-MFM), each second (FIR-MAP-MFM-2) and sixth (FIR-MAP-MFM-6) projection in the initial step and iteration process. Each method consists of mean value in ROI (mean) and standard deviation (std). Additionally, results of all volunteers are presented in the form of mean/std.

	FIR-MAP-MFM			FIR-MAP-MFM-2			FIR-MAP-MFM-6		
	WM	GM	CSF	WM	GM	CSF	WM	GM	CSF
Mean	710	1407	4016	702	1405	3962	703	1372	3664
Std	31	115	469	40	114	464	64	135	424
Mean/Std	23	12	9	18	12	9	11	10	9

## Supplementary Materials

The following are available online at https://www.mdpi.com/1424-8220/19/24/5371/s1: Source codes of all scripts written in Matlab and manual with full explanation of steps required in application of scripts.

## Appendix A

**Table A1:** Results of T_1_ values (in ms) of ROI analysis for initial model MFM calculated for 150 iterations of FIR-MAP by taking all projections (FIR-MAP-MFM), each second (FIR-MAP-MFM-2) and sixth (FIR-MAP-MFM-6) projection in initial step and iteration process. Each method consists of mean value in ROI (mean) and standard deviation (std). Additionally, results of all volunteers are presented in the form of mean/std.

	V1	V2	V3	V4	V5	V6	V7	Mean	Mean/Std
White Matter (WM)									
FIR-MAP-IFM	733	700	740	685	677	725	778	720	9
	82	78	68	77	81	100	66	79	
FIR-MAP-MFM	725	706	722	674	684	705	755	710	23
	32	28	30	37	34	35	24	31	
IR-MAP	738	706	740	688	679	726	779	722	9
	82	76	68	78	82	99	63	78	
REF	734	709	712	695	693	698	744	712	32
	16	42	21	31	17	17	11	22	
**Grey Matter (GM)**									
FIR-MAP-IFM	1435	1407	1454	1447	1426	1401	1420	1427	9
	131	84	160	207	273	89	170	159	
FIR-MAP-MFM	1415	1365	1438	1419	1415	1401	1394	1407	12
	89	63	113	154	246	45	94	115	
IR-MAP	1447	1405	1459	1453	1429	1400	1430	1432	9
	127	85	159	207	286	90	178	162	
REF	1436	1385	1402	1378	1401	1400	1409	1402	12
	94	98	158	147	192	58	71	117	
**Cerebrospinal Fluid (CSF)**									
FIR-MAP-IFM	4598	4508	4466	4043	4364	4056	4343	4340	7
	586	633	702	450	874	304	667	602	
FIR-MAP-MFM	4295	4106	4142	3772	4083	3704	4009	4016	9
	424	559	562	344	738	167	486	469	
IR-MAP	4603	4585	4473	3990	4387	4057	4360	4351	7
	646	669	720	506	925	364	726	651	
REF	4296	4082	3877	3924	4061	3236	3878	3908	7
	494	620	700	348	911	249	564	555	

**Table A2:** Results of T_1_ values (in ms) of ROI analysis for initial model MFM calculated for 150 iterations and initial model IFM calculated for 30 iterations of FIR-MAP by taking each second (FIR-MAP-MFM-2, FIR-MAP-IFM-2) and sixth (FIR-MAP-MFM-6, FIR-MAP-IFM-6) projection in iteration process. Each method consists of mean value in ROI (each upper row) and standard deviation (each lower row). Additionally, results of all volunteers are presented in the mean value and corresponding mean/std is calculated.

	V1	V2	V3	V4	V5	V6	V7	Mean	Mean/Std
White Matter (WM)									
FIR-MAP-MFM-2	704	699	710	673	671	721	731	701	17
	34	49	37	47	39	44	35	41	
FIR-MAP-IFM-2	721	697	737	683	674	732	773	717	9
	81	79	68	75	82	100	66	79	
FIR-MAP-MFM-6	674	743	679	691	687	738	696	701	10
	72	58	78	73	61	55	81	68	
FIR-MAP-IFM-6	694	710	718	676	662	751	756	710	9
	81	80	67	75	85	105	70	80	
**Grey Matter (GM)**									
FIR-MAP-MFM-2	1426	1368	1409	1403	1401	1414	1393	1402	12
	89	67	105	153	241	58	92	115	
FIR-MAP-IFM-2	1444	1409	1446	1439	1421	1406	1417	1426	9
	134	85	164	206	274	92	173	161	
FIR-MAP-MFM-6	1398	1348	1359	1347	1336	1394	1405	1370	11
	104	83	175	155	218	60	104	128	
FIR-MAP-IFM-6	1447	1389	1453	1422	1369	1400	1432	1416	9
	132	84	173	206	267	93	174	161	
**Cerebrospinal Fluid (CSF)**									
FIR-MAP-MFM-2	4298	4038	4088	3743	3965	3623	3956	3959	9
	467	551	538	314	689	155	496	459	
FIR-MAP-IFM-2	4602	4504	4467	4047	4337	4035	4330	4332	7
	593	628	695	443	864	287	668	597	
FIR-MAP-MFM-6	3940	3778	3902	3506	3749	3537	3722	3733	9
	343	513	511	181	657	203	450	408	
FIR-MAP-IFM-6	4575	4480	4454	4034	4311	4024	4288	4309	7
	562	623	672	429	869	312	654	589	

**Table A3:** Results of T_1_ values (in ms) of ROI analysis for initial model MFM calculated for 150 iterations of FIR-MAP by taking each second (FIR-MAP-MFM-2) and sixth (FIR-MAP-MFM-6) projection in initial step and iteration process. Each method consists of mean value in ROI (each upper row) and standard deviation (each lower row). Additionally, results of all volunteers are presented in the mean value and corresponding mean/std is calculated.

	V1	V2	V3	V4	V5	V6	V7	Mean	Mean/Std
White Matter (WM)									
FIR-MAP-MFM-2	705	698	708	675	670	725	731	702	18
	31	50	34	47	39	44	36	40	
FIR-MAP-MFM-6	668	740	681	687	700	745	702	703	11
	66	55	66	68	56	55	83	64	
**Grey Matter (GM)**									
FIR-MAP-MFM-2	1431	1374	1407	1406	1401	1418	1398	1405	12
	93	72	101	153	233	55	92	114	
FIR-MAP-MFM-6	1395	1349	1370	1349	1344	1392	1406	1372	10
	117	90	201	156	217	61	104	135	
**Cerebrospinal Fluid (CSF)**									
FIR-MAP-MFM-2	4305	4042	4078	3756	3974	3620	3960	3962	9
	455	563	527	324	692	190	495	464	
FIR-MAP-MFM-6	3931	3653	3810	3480	3668	3446	3662	3664	9
	327	566	535	214	636	252	440	424	
