# Supplementary material for: Improvement of Fast Model-Based Acceleration of Parameter Look-Locker T1 Mapping
Source: Sensors (Basel). 2019 Dec 5;19(24):5371. doi: 10.3390/s19245371 (PMC6960582; doi:10.3390/s19245371)
Supplement: Supplementary file 1 [file sensors-19-05371-s001.zip › FIR-MAP/manual/initial_guess.html]

Function initial\_guess 

# Function initial\_guess

Calculates initial guess of T1s, M0, M0s

## Contents

- Input
- Output
- Copyrights

## Input

- cons\_model\_sos - combined consistant model in image space for all coils of size np x nr x nr
- maske - image mask of size nr x nr
- nr - number of rows
- first\_T1s - initial value of T1s for whole image

## Output

- M0s - first initial M0s image
- M0 - first initial M0 image
- T1s - first initial T1s image

## Copyrights

(C) All rights reserved.

The code may be used free of charge for non-commercial and educational purposes, the only requirement is that this text is preserved within the derivative work. For any other purpose you must contact the authors for permission. This code may not be redistributed without written permission from the authors.

ABOUT: This software implements basic functionalities of the FIR-MAP algorithm

IMPORTANT: If you use this software you should cite the following in any resulting publication: [1] Michal Staniszewski and Uwe Klose. Improvements of Fast Model-based Acceleration of Parameter Look-Locker T1 Mapping

```
function [ T1s, M0s, M0 ] = initial_guess( maske, cons_model_sos, nr, first_T1s )

    T1s = zeros(nr,nr);
    M0s = zeros(nr,nr);
    M0 = zeros(nr,nr);
    T1s(maske>0) = first_T1s;

    for b=1:nr
        ok_tmp = cons_model_sos(:,:,b); % take all magnetization curves
        fprintf(['Iter #',num2str(0),'. Compute first M0 M0s. Col #',num2str(b),'\n']);
        for a=1:nr
            if (logical(maske(a,b)))
                ok = ok_tmp(:,a);
                M0s(a,b) = max(ok);
                M0(a,b) = -min(ok);
            end
        end
    end

end
```

Published with MATLAB® R2016b
