# Supplementary material for: Improvement of Fast Model-Based Acceleration of Parameter Look-Locker T1 Mapping
Source: Sensors (Basel). 2019 Dec 5;19(24):5371. doi: 10.3390/s19245371 (PMC6960582; doi:10.3390/s19245371)
Supplement: Supplementary file 1 [file sensors-19-05371-s001.zip › FIR-MAP/manual/read_projections_step.html]

Function read\_projections\_step 

# Function read\_projections\_step

Reads original projections and preparing data for initial starting model

## Contents

- Input
- Output
- Copyrights

## Input

- timeInterval - time interval of data acquisition
- string - storing path and localization of original projections to be read

## Output

- n\_size - data size [np - number of projections, nc - number of coils, nr - number of rows]
- init\_image\_avg - MFM model used in first model of size nr x nr x nc
- recon\_grog - original projections in k-space of size nr x nr x nc x np
- meanPhase - mean phase of size nc x nr x nr calculated according to Tran-Gia et. al. (2014)
- recon\_grog\_kspace - IFM model in k-space used as consistant model of size nr x nr x nc x np

## Copyrights

(C) All rights reserved.

The code may be used free of charge for non-commercial and educational purposes, the only requirement is that this text is preserved within the derivative work. For any other purpose you must contact the authors for permission. This code may not be redistributed without written permission from the authors.

ABOUT: This software implements basic functionalities of the FIR-MAP algorithm

IMPORTANT: If you use this software you should cite the following in any resulting publication: [1] Michal Staniszewski and Uwe Klose. Improvements of Fast Model-based Acceleration of Parameter Look-Locker T1 Mapping

```
function [n_size, init_image_avg, recon_grog, meanPhase, recon_grog_kspace] = read_projections_step( string, timeInterval )

    fprintf('Load and permute rawData for %s\n', string)
    eval(string); % read data
    rawSegm=permute(rawSegm,[2 3 1 4]); % permute to have dimensions  coils x projections x segments x readout points
    singleRawSegmOrig = squeeze(rawSegm(:,:,1,:)); % take only first segment of data of dimensions coils x projections x readout points
    proj_step = 1; % take each point
    singleRawSegm = singleRawSegmOrig(:,1:proj_step:999,:);

    np_orig = size(singleRawSegmOrig,2); % nb of original projections

    fprintf('Create rad traj')
    traj=createRadTrajGR(np_orig,size(singleRawSegm,3)); % dim 999 x 256 complex double <----- .p

    % recon to store all gridded projectories
    np = size(singleRawSegm,2); % number of projections
    nc = size(singleRawSegm,1); % number of coils
    nr = size(singleRawSegm,3); % numberb of image rows
    n_size = [np, nc, nr];

    radNyq_orig = round(nr/2*pi/2); % last 200 projections
    radNyq = round(nr/2*pi/2/proj_step);
    grog_traj = GROG(singleRawSegm(:,np-radNyq+1:np,:),traj(np_orig-radNyq_orig+1:proj_step:np_orig,:));
    meanPhase = angle(ifft2d(grog_traj));

    recon_grog = zeros(nr, nr, nc, np); % to store original projections in k-space
    recon_grog_kspace = zeros(nr, nr, nc, np); % to store projections in k-space from IFM first model

    nb_presence = zeros(nr,nr,nc); % nb of non-zero elements present in given pixel
    init_image_coils = zeros(nr,nr,nc); % final init images for all coils

    proj_start = 1; % iterate from 1 up to np projection
    proj_stop = np;
    for a=proj_start:proj_stop
        fprintf('Grid all trajectiories for each projection -> #%d\n', a)
        calc_grog = GROG(singleRawSegm(:,a,:),traj((proj_step*(a - 1) + 1),:)); % dim 4 x 256 x 256 complex double -> k-space
        calc_grog = permute(calc_grog, [2 3 1]);
        recon_grog(:,:,:,a) = calc_grog;

        init_image_coils = init_image_coils + calc_grog; % calc values of all k-space projections 4x256x256
        tmp_logic = calc_grog~=0; % find non-zero elements in k-space
        nb_presence = nb_presence + tmp_logic; % sum number of non-zero elements
    end

    % take into account number of non-zero elements
    nb_presence(nb_presence == 0) = 1; %
    disp('Combine first model for each coil')
    init_image_avg_tmp = init_image_coils./nb_presence; % k-space
    init_image_avg = fftshift(ifft(ifft(fftshift(init_image_avg_tmp),[],1),[],2)); % image-space initial MFM

    % interpolated for each coil
    for coil = 1:nc
        fprintf('Rebuilding #%d single coil Kspace\n', coil)
        kspace = squeeze(recon_grog(:,:,coil,:));
        for a=1:nr
            for b = 1:nr
                ok=squeeze(kspace(a,b,:)).';
                if (~(max(real(ok)) == 0 && min(real(ok)) == 0 && max(imag(ok)) == 0 && min(imag(ok)) == 0)) % if at least one element different than 0 in projection
                    [~,ind,~] = find(ok);
                    if (size(ind,2) > 1) % at least two elements in projection
                        temp = interp1(timeInterval(ind),ok(ind),timeInterval,'pchip');
                        kspace(a,b,:) = temp;
                    else
                        kspace(a,b,:) = ok(ind);
                    end
                end
            end
        end
        recon_grog_kspace(:,:,coil,:) = kspace;
    end

end
```

Published with MATLAB® R2016b
