# Supplementary material for: Improvement of Fast Model-Based Acceleration of Parameter Look-Locker T1 Mapping
Source: Sensors (Basel). 2019 Dec 5;19(24):5371. doi: 10.3390/s19245371 (PMC6960582; doi:10.3390/s19245371)
Supplement: Supplementary file 1 [file sensors-19-05371-s001.zip › FIR-MAP/manual/compute_T1_M0.html]

Function compute\_T1\_M0 

# Function compute\_T1\_M0

Computes first step of pixel-wise fitting of 3 parameters

## Contents

- Input
- Output
- Copyrights

## Input

- n\_size - data size [np - number of projections, nc - number of coils, nr - number of rows]
- cons\_model\_sos - combined consistant model in image space for all coils of size np x nr x nr
- maske - image mask of size nr x nr
- timeInterval - time interval of data acquisition
- n\_iter - number of current iteration
- T1s - T1s image values from previous iteration
- M0 - M0 image values from previous iteration
- M0s - M0s image values from previous iteration

## Output

- T1s - image values of T1s
- M0 - image values of M0
- M0s - image values of M0s
- k\_factor - image values of relation M0/M0s

## Copyrights

(C) All rights reserved.

The code may be used free of charge for non-commercial and educational purposes, the only requirement is that this text is preserved within the derivative work. For any other purpose you must contact the authors for permission. This code may not be redistributed without written permission from the authors.

ABOUT: This software implements basic functionalities of the FIR-MAP algorithm

IMPORTANT: If you use this software you should cite the following in any resulting publication: [1] Michal Staniszewski and Uwe Klose. Improvements of Fast Model-based Acceleration of Parameter Look-Locker T1 Mapping

```
function [ T1s, M0, M0s, k_factor] = compute_T1_M0( n_size, cons_model_sos, maske, timeInterval, n_iter, T1s, M0, M0s )

    nr = n_size(3); % number of image rows
    k_factor = zeros(nr,nr);
    x = timeInterval';

    % iterate in parallel version
    parfor b=1:nr
        ok_tmp = cons_model_sos(:,:,b); % magnetization curves
        fprintf(['Iter #',num2str(n_iter),'. Compute T1* M0 M0s. Reconstructing col #',num2str(b),'\n']);
        for a=1:nr
            if (logical(maske(a,b)))
                ok = ok_tmp(:,a);
                [ypos,pos] = min(ok);
                ok(1:pos(1)) = 2*ypos - ok(1:pos(1)); % shift curve
                init_guess = [M0s(a,b) T1s(a,b) M0(a,b)];
                expfn = @(p,xd) p(1)-(p(3)+p(1))*exp(-xd/p(2));  % define M(t)
                try
                    pfit = nlinfit(x,ok,expfn,init_guess); % faster non-linear regression
                catch
                    errfn = @(p) sum((expfn(p,x)-ok).^2);  % define sum-squared error
                    pfit = fminsearch(errfn, init_guess);     %run the minimizer - more stable in case of problems
                end
                T1s(a,b) = pfit(2);
                M0s(a,b) = pfit(1);
                M0(a,b) = pfit(3);
                k_factor(a,b) = M0(a,b)/M0s(a,b);
            end
        end
    end
end
```

Published with MATLAB® R2016b
