# Supplementary material for: Improvement of Fast Model-Based Acceleration of Parameter Look-Locker T1 Mapping
Source: Sensors (Basel). 2019 Dec 5;19(24):5371. doi: 10.3390/s19245371 (PMC6960582; doi:10.3390/s19245371)
Supplement: Supplementary file 1 [file sensors-19-05371-s001.zip › FIR-MAP/manual/recon_main_T1map.html]

Script recon\_main\_T1map.m 

# Script recon\_main\_T1map.m

Main running script of the FIR-MAP algorithm

## Contents

- First run
- Copyrights
- load list of indivudual measurement IDs generated by the scanner
- load raw data
- compute first model from projections and first consistant model
- first initial guest
- compute T1, M0s and M0 for first model within first step of pixel-wise fitting for first iteration
- compute second step of pixel-wise fitting for first iteration
- perform iteration process
- compute model and consistant model for next iteration
- compute T1, M0s and M0 for model within first step of pixel-wise fitting for next iteration
- compute second step of pixel-wise fitting for next iteration
- display results and optionally get rid off outliers

## First run

1) The Matlab code was tested in version 2015 and 2016. Source code uses standard Matlab libraries and few functions provided within Tran-Gia et al. work located in 'p' folder. 2) Download source code and setup basic parameters in recon\_main\_T1map.m: - prevPath='../'; % main localization of raw folder from Tran-Gia et. al (2014) reference data - startingV = 1; % starting number of volunteer - stopingV = 8; % ending number of volunteer - n\_iter = 30; % nb of iterations - interpol = 1; % type of first model 0 - MFM or -1- IFM - first\_T1s = 1000; % initial value of T1s for MFM - np = 999; % number of projections 3) Download raw data and place them in 'raw' folder. Notice that 'prevPath' defines where 'raw' folder is located with respect to recon\_main\_T1map.m. In order to use reference data it is necessary to download raw data provided by Tran-Gia et al. basing on the study "Model-Based Acceleration of Look-Locker T1 Mapping" PlosOne 2014. 4) Run recon\_main\_T1map.m

## Copyrights

(C) All rights reserved.

The code may be used free of charge for non-commercial and educational purposes, the only requirement is that this text is preserved within the derivative work. For any other purpose you must contact the authors for permission. This code may not be redistributed without written permission from the authors.

ABOUT: This software implements basic functionalities of the FIR-MAP algorithm

IMPORTANT: If you use this software you should cite the following in any resulting publication: [1] Michal Staniszewski and Uwe Klose. Improvements of Fast Model-based Acceleration of Parameter Look-Locker T1 Mapping

```
addpath('scripts') % scripts for the FIR-MAP algorithm
addpath('p') % p files for trajectories and GROG
prevPath='../'; % main localization of raw folder from Tran-Gia et. al (2014) reference data
```

## load list of indivudual measurement IDs generated by the scanner

```
load([prevPath,'raw/MIDs.mat']);
startingV = 1; % starting number of volunteer
stopingV = 1; % ending number of volunteer
n_iter = 3; % nb of iterations
interpol = 1; % type of first model 0 - MFM or -1- IFM
first_T1s = 1000; % initial value of T1s for MFM
np = 999; % number of projections

for V=startingV:stopingV
```

## load raw data

```
    iter_time = zeros(n_iter,1); % stores time of each iteration

    % load paramaters and compute Inversion Times
    load([prevPath,'raw/paramsVall.mat'])
    timeInterval=params.firstTI:params.TR:params.firstTI+(np-1)*params.TR; % time interval of data acquisition

    % load image mask
    string=(['load(''',prevPath,'raw/maskV',num2str(V),'.mat'')']);
    eval(string);

    % load 'rawdata' for one volunteer and perform first model evaluation
    string =(['load(''',prevPath,'raw/rawSegmV',num2str(V),'.mat'')']);
    [n_size, init_image_avg, recon_grog, meanPhase, recon_grog_first] = read_projections_step( string, timeInterval );

    % calculate k-space mask
    mask_k=logical(abs(squeeze(sum(recon_grog,4))));
```

## compute first model from projections and first consistant model

```
    tic % first iteration
    [ cons_model_coil, cons_model_sos] = compute_first_model( n_size, init_image_avg, timeInterval, recon_grog, meanPhase, first_T1s, interpol, recon_grog_first, mask_k);
    iter_time(1,1) = toc;

    np = n_size(1); % number of projections
    nc = n_size(2); % number of coils
    nr = n_size(3); % number of rows
    x = timeInterval;
    diff = zeros(n_size(1),n_iter-1);
    T1_map_iter = zeros(n_iter, nr,nr);
```

## first initial guest

```
    [ T1s, M0s, M0 ] = initial_guess( maske, cons_model_sos, nr, first_T1s );
```

## compute T1, M0s and M0 for first model within first step of pixel-wise fitting for first iteration

```
    tic
    [ T1s, M0, M0s, k_factor] = compute_T1_M0( n_size, cons_model_sos, maske, timeInterval, 1, T1s, M0, M0s );
    iter_time(1,2) = toc;
```

## compute second step of pixel-wise fitting for first iteration

```
    tic
    [ M0s_prodsens, M0_new, T1_map, exp_factor ] = compute_M0_coil( n_size, cons_model_coil, maske, timeInterval, 1, T1s, M0s, M0, k_factor);
    T1_map_iter(1,:,:) = T1_map;
    iter_time(1,3) = toc;
    iter_time(1,4) = iter_time(1,1) + iter_time(1,2) + iter_time(1,3);
```

## perform iteration process

```
    for iter=1:(n_iter-1)
```

## compute model and consistant model for next iteration

```
        tic
        fprintf('********* Iteration #%d ************\n', iter + 1)
        [ cons_model_coil, cons_model_sos, diff(:,iter) ] = compute_model( n_size, recon_grog, M0s_prodsens, M0_new, iter+1, exp_factor, meanPhase, mask_k);
        iter_time(iter+1,1) = toc;
```

## compute T1, M0s and M0 for model within first step of pixel-wise fitting for next iteration

```
        tic
        [ T1s, M0, M0s, k_factor] = compute_T1_M0( n_size, cons_model_sos, maske, timeInterval, iter+1, T1s, M0, M0s );
        iter_time(iter+1,2) = toc;
```

## compute second step of pixel-wise fitting for next iteration

```
        tic
        [ M0s_prodsens, M0_new, T1_map, exp_factor ] = compute_M0_coil( n_size, cons_model_coil, maske, timeInterval, iter+1, T1s, M0s, M0, k_factor);
        T1_map_iter(iter+1,:,:) = T1_map;
        iter_time(iter+1,3) = toc;
        iter_time(iter+1,4) = iter_time(iter+1,1) + iter_time(iter+1,2) + iter_time(iter+1,3);
```

```
    end
```

## display results and optionally get rid off outliers

```
    figure
    T1_map(T1_map>6000) = 0;
    T1_map(T1_map<0) = 0;
    imagesc(T1_map)
```

```
end
```

Published with MATLAB® R2016b
