# Supplementary material for: Improvement of Fast Model-Based Acceleration of Parameter Look-Locker T1 Mapping
Source: Sensors (Basel). 2019 Dec 5;19(24):5371. doi: 10.3390/s19245371 (PMC6960582; doi:10.3390/s19245371)
Supplement: Supplementary file 1 [file sensors-19-05371-s001.zip › FIR-MAP/manual/calculateSos.html]

Function calculateSos 

# Function calculateSos

Computes combined image for all coils calculated according to Tran-Gia et. al. (2014)

## Contents

- Input
- Output
- Copyrights

## Input

- meanPhase - mean phase of size nc x nr x nr calculated according to Tran-Gia et. al. (2014)
- cons\_model\_coil - consistant model in image space for whole dataset of size np x nr x nr x nc

## Output

- M\_sos - combined consistant model in image space for all coils of size np x nr x nr

## Copyrights

(C) All rights reserved.

The code may be used free of charge for non-commercial and educational purposes, the only requirement is that this text is preserved within the derivative work. For any other purpose you must contact the authors for permission. This code may not be redistributed without written permission from the authors.

ABOUT: This software implements basic functionalities of the FIR-MAP algorithm

IMPORTANT: If you use this software you should cite the following in any resulting publication: [1] Michal Staniszewski and Uwe Klose. Improvements of Fast Model-based Acceleration of Parameter Look-Locker T1 Mapping

```
function M_sos = calculateSos(meanPhase, cons_model_coil)

    nc = size(cons_model_coil,4); % number of coils
    np = size(cons_model_coil,1); % number of projections
    nr = size(cons_model_coil,2); % number of image rows
    O_nc=zeros(np,nr,nr);

    % iterate for all coils
    for c=1:nc
        % build imspace for each coil
        fprintf('Calculate SoS for coil#%d\n',c)
        M_Re=cons_model_coil(:,:,:,c);
        temp_meanPhase = meanPhase(c,:,:);
        temp_prod = M_Re.*exp(-1i*temp_meanPhase);
        M_Re = real(temp_prod); % (6) Tran-Gia et. al. (2014)
        % combine all coils
        O_nc=O_nc+sign(M_Re).*abs(M_Re).^2; % (8) Tran-Gia et. al. (2014)
    end
    M_sos=sign(O_nc).*sqrt(abs(O_nc)); % (7) Tran-Gia et. al. (2014)

end
```

Published with MATLAB® R2016b
