# Supplementary material for: Improvement of Fast Model-Based Acceleration of Parameter Look-Locker T1 Mapping
Source: Sensors (Basel). 2019 Dec 5;19(24):5371. doi: 10.3390/s19245371 (PMC6960582; doi:10.3390/s19245371)
Supplement: Supplementary file 1 [file sensors-19-05371-s001.zip › FIR-MAP/manual/compute_model.html]

Function compute\_model 

# Function compute\_model

Computes model in image space and consistant model for each projection

## Contents

- Input
- Output
- Copyrights

## Input

- n\_size - data size [np - number of projections, nc - number of coils, nr - number of rows]
- recon\_grog - original projections in k-space of size nr x nr x nc x np
- M0s\_prodsens - M0s for all coils of size nr x nr x nc
- M0\_new - M0 for all coils of size nr x nr x nc
- n\_iter - number of current iteration
- exp\_factor - calculated exponential factor in the form of exp(-TI/T1s)
- meanPhase - mean phase of size nc x nr x nr calculated according to Tran-Gia et. al. (2014)
- mask\_k - k-space circular mask

## Output

- cons\_model\_coil - consistant model in image space for whole dataset of size np x nr x nr x nc
- cons\_model\_sos - combined consistant model in image space for all coils of size np x nr x nr
- diff - difference value used in evaluation of termination criterion

## Copyrights

(C) All rights reserved.

The code may be used free of charge for non-commercial and educational purposes, the only requirement is that this text is preserved within the derivative work. For any other purpose you must contact the authors for permission. This code may not be redistributed without written permission from the authors.

ABOUT: This software implements basic functionalities of the FIR-MAP algorithm

IMPORTANT: If you use this software you should cite the following in any resulting publication: [1] Michal Staniszewski and Uwe Klose. Improvements of Fast Model-based Acceleration of Parameter Look-Locker T1 Mapping

```
function [ cons_model_coil, cons_model_sos, diff ] = compute_model( n_size, recon_grog, M0s_prodsens, M0_new, n_iter, exp_factor, meanPhase, mask_k)

    np = n_size(1); % number of projections
    nc = n_size(2); % number of coils
    nr = n_size(3); % number of image rows
    diff = zeros(nc,1);
    cons_model_coil = zeros(nr,nr,nc,np); %image

    % iterate for all projections
    for a = 1:np
        fprintf('Iter #%d. Calculate consistant model for each projection and coil. Projection -> #%d\n', n_iter, a)
        M_t = M0s_prodsens - (M0_new + M0s_prodsens) .* exp_factor(:,:,a); % magnetization curve M(t) in image space
        projection_model = fftshift(fft(fft(fftshift(M_t),[],1),[],2)); % go back to k-space
        single_proj_raw = recon_grog(:,:,:,a); % in k-space single projection from raw data

        % calculate corresponding mask and reinsert initial projections
        mask=logical(abs(single_proj_raw)); % creating mask for single projection
        diff(a)=mean(abs(abs(projection_model(mask))-abs(single_proj_raw(mask)))); % calculating difference for termination criterion
        projection_model(mask)=single_proj_raw(mask); % reinserting single projection
%         projection_model(~mask_k) = 0; % optional k-space mask

        % calculate consistant model
        projection_model_coil=fftshift(ifft(ifft(fftshift(projection_model),[],1),[],2)); % move to image space
        cons_model_coil(:,:,:,a) = projection_model_coil;
    end

    % calculate combined model
    cons_model_coil = shiftdim(cons_model_coil,3);
    cons_model_sos = calculateSos(meanPhase, cons_model_coil);

end
```

Published with MATLAB® R2016b
