# Supplementary material for: Improvement of Fast Model-Based Acceleration of Parameter Look-Locker T1 Mapping
Source: Sensors (Basel). 2019 Dec 5;19(24):5371. doi: 10.3390/s19245371 (PMC6960582; doi:10.3390/s19245371)
Supplement: Supplementary file 1 [file sensors-19-05371-s001.zip › FIR-MAP/manual/compute_M0_coil.html]

Function compute\_M0\_coil 

# Function compute\_M0\_coil

Computes second step of pixel-wise fitting of 1 parameter for coils

## Contents

- Input
- Output
- Copyrights

## Input

- n\_size - data size [np - number of projections, nc - number of coils, nr - number of rows]
- cons\_model\_coil - consistant model in image space for whole dataset of size np x nr x nr x nc
- maske - image mask of size nr x nr
- timeInterval - time interval of data acquisition
- n\_iter - number of current iteration
- T1s - T1s image values from first step for T1 map
- M0 - M0 image values from first step for T1 map
- M0s - M0s image values from first step for T1 map
- k\_factor - image values of relation M0/M0s

## Output

- M0s\_prodsens - M0s for all coils of size nr x nr x nc
- M0\_new - M0 for all coils of size nr x nr x nc
- exp\_factor - calculated exponential factor in the form of exp(-TI/T1s)
- T1\_map - image values of T1

## Copyrights

(C) All rights reserved.

The code may be used free of charge for non-commercial and educational purposes, the only requirement is that this text is preserved within the derivative work. For any other purpose you must contact the authors for permission. This code may not be redistributed without written permission from the authors.

ABOUT: This software implements basic functionalities of the FIR-MAP algorithm

IMPORTANT: If you use this software you should cite the following in any resulting publication: [1] Michal Staniszewski and Uwe Klose. Improvements of Fast Model-based Acceleration of Parameter Look-Locker T1 Mapping

```
function [ M0s_prodsens, M0_new, T1_map, exp_factor ] = compute_M0_coil( n_size, cons_model_coil, maske, timeInterval, n_iter, T1s, M0s, M0, k_factor)

    np = n_size(1); % number of projections
    nc = n_size(2); % number of coils
    nr = n_size(3); % number of image rows
    x = timeInterval';

    M0s_prodsens = zeros(nr,nr,nc);
    M0_new = zeros(nr,nr,nc);
    exp_factor = zeros(nr,nr,np);

    % iterate for all coils
    M0s_prodsens_coil = zeros(nr,nr);
    for c=1:1:nc
        ok_tmp = cons_model_coil(:,:,:,c); % magnetization curves
        for b=1:nr
            ok_coil = ok_tmp(:,:,b);
            fprintf('Iter #%d. Include multiple receiver coil for coil #%d. Row -> #%d\n', n_iter, c, b)
            for a=1:nr
                if (logical(maske(a,b)))
                    ok = ok_coil(:,a);
                    y = real(ok);
                    y2 = imag(ok);
                    E = (1-(k_factor(a,b)+1)*exp(-x/T1s(a,b)));
                    pfit_real  = E\y; % linear one parameter fitting for real part
                    pfit_imag  = E\y2; % linear one parameter fitting for real part
                    M0s_prodsens_coil(a,b) = complex(pfit_real(1),pfit_imag(1));
                    exp_factor(a,b,:) = exp(-timeInterval/T1s(a,b));
                end
            end
        end
        M0s_prodsens(:,:,c) = M0s_prodsens_coil;
        M0_new(:,:,c) = M0s_prodsens_coil.*k_factor;
    end

    T1_map = T1s .* k_factor;
end
```

Published with MATLAB® R2016b
