# Supplementary material for: Improvement of Fast Model-Based Acceleration of Parameter Look-Locker T1 Mapping
Source: Sensors (Basel). 2019 Dec 5;19(24):5371. doi: 10.3390/s19245371 (PMC6960582; doi:10.3390/s19245371)
Supplement: Supplementary file 1 [file sensors-19-05371-s001.zip › FIR-MAP/manual/compute_first_model.html]

Function compute\_first\_model 

# Function compute\_first\_model

Computes first model MFM or IFM depending on choice and first consistant model for each projection

## Contents

- Input
- Output
- Copyrights

## Input

- n\_size - data size [np - number of projections, nc - number of coils, nr - number of rows]
- init\_image\_avg - MFM model used in first model of size nr x nr x nc
- timeInterval - time interval of data acquisition
- recon\_grog - original projections in k-space of size nr x nr x nc x np
- meanPhase - mean phase of size nc x nr x nr calculated according to Tran-Gia et. al. (2014)
- first\_T1s - first initial value of T1s for MFM
- interpol - choice of first model - 0 - MFM, 1 - IFM
- recon\_grog\_first - IFM model in k-space used as consistant model of size nr x nr x nc x np
- mask\_k - k-space circular mask

## Output

- cons\_model\_coil - consistant model in image space for whole dataset of size np x nr x nr x nc
- cons\_model\_sos - combined consistant model in image space for all coils of size np x nr x nr

## Copyrights

(C) All rights reserved.

The code may be used free of charge for non-commercial and educational purposes, the only requirement is that this text is preserved within the derivative work. For any other purpose you must contact the authors for permission. This code may not be redistributed without written permission from the authors.

ABOUT: This software implements basic functionalities of the FIR-MAP algorithm

IMPORTANT: If you use this software you should cite the following in any resulting publication: [1] Michal Staniszewski and Uwe Klose. Improvements of Fast Model-based Acceleration of Parameter Look-Locker T1 Mapping

```
function [ cons_model_coil, cons_model_sos] = compute_first_model( n_size, init_image_avg, timeInterval, recon_grog, meanPhase, first_T1s, interpol, recon_grog_first, mask_k)

    np = n_size(1); % number of projections
    nc = n_size(2); % number of coils
    nr = n_size(3); % number of image rows
    cons_model_coil = zeros(nr,nr,nc,np);
    exp_factor = exp(-timeInterval./first_T1s); % exponential factor in the form of exp(-TI/T1s)

    % iterate for all projections
    for a = 1:np
        fprintf('calculate first model and consistant model for each projection and coil. Projection -> #%d\n', a)

        if interpol
            % for interpolated first model IFM
            projection_model = recon_grog_first(:,:,:,a); % use data calculated in interpolation process
            projection_model_coil=fftshift(ifft(ifft(fftshift(projection_model),[],1),[],2));
            cons_model_coil(:,:,:,a) = projection_model_coil;
        else
            % for mean first model MFM
            M_t = init_image_avg - 2*init_image_avg .* exp_factor(a); % magnetization curve M(t) in image
            projection_model = fftshift(fft(fft(fftshift(M_t),[],1),[],2)); % go back to k-space
            single_proj_raw = recon_grog(:,:,:,a); % in k-space single projection from raw data

            % calculate corresponding mask and reinsert initial projections
            mask=logical(abs(single_proj_raw));
            projection_model(mask)=single_proj_raw(mask);
%             projection_model(~mask_k) = 0; % optional k-space mask

            % calculate consistant model
            projection_model_coil=fftshift(ifft(ifft(fftshift(projection_model),[],1),[],2)); % move to image space
            cons_model_coil(:,:,:,a) = projection_model_coil;
        end
    end

    % calculate combined model
    cons_model_coil = shiftdim(cons_model_coil,3);
    cons_model_sos = calculateSos(meanPhase, cons_model_coil);

end
```

Published with MATLAB® R2016b
